# Knowledge brokering for healthy aging: a scoping review of potential approaches

**DOI:** 10.1186/s13012-016-0504-5

**Published:** 2016-10-19

**Authors:** Dwayne Van Eerd, Kristine Newman, Ryan DeForge, Robin Urquhart, Evelyn Cornelissen, Katie N. Dainty

**Affiliations:** 1 Institute for Work and Health, 481 University Avenue, Suite 800, Toronto, Ontario Canada M5G 2E9; 2 School of Public Health and Health Systems, University of Waterloo, 200 University Avenue West, Waterloo, Ontario Canada N2L 3G1; 3 Daphne Cockwell School of Nursing, Faculty of Community Services, Ryerson University, 350 Victoria Street, Toronto, Ontario Canada M5B 2K3; 4 World Health Innovation Network, Odette School of Business, University of Windsor, 401 Sunset Avenue, Windsor, Ontario Canada N9B 3P4; 5 Department of Surgery, Dalhousie University, 6299 South Street, Halifax, Nova Scotia Canada B3H 4R2; 6 Department of Family Practice, Faculty of Medicine, University of British Columbia, 2312 Pandosy Street, Kelowna, British Columbia Canada V1Y 1T3; 7 Li Ka Shing Knowledge Institute, St. Michael’s Hospital, 209 Victoria Street, Toronto, Ontario Canada M5B 1T8; 8 Institute of Health Policy, Management and Evaluation, University of Toronto, 155 College Street, Toronto, Ontario Canada M5T 3M6

**Keywords:** Knowledge transfer, Knowledge broker, Healthy aging

## Abstract

**Background:**

Developing a healthcare delivery system that is more responsive to the future challenges of an aging population is a priority in Canada. The World Health Organization acknowledges the need for knowledge translation frameworks in aging and health. Knowledge brokering (KB) is a specific knowledge translation approach that includes making connections between people to facilitate the use of evidence. Knowledge gaps exist about KB roles, approaches, and guiding frameworks. The objective of the scoping review is to identify and describe KB approaches and the underlying conceptual frameworks (models, theories) used to guide the approaches that could support healthy aging.

**Methods:**

Literature searches were done in PubMed, EMBASE, PsycINFO, EBM reviews (Cochrane Database of systematic reviews), CINAHL, and SCOPUS, as well as Google and Google Scholar using terms related to knowledge brokering. Titles, abstracts, and full reports were reviewed independently by two reviewers who came to consensus on all screening criteria. Documents were included if they described a KB approach and details about the underlying conceptual basis. Data about KB approach, target stakeholders, KB outcomes, and context were extracted independently by two reviewers.

**Results:**

Searches identified 248 unique references. Screening for inclusion revealed 19 documents that described 15 accounts of knowledge brokering and details about conceptual guidance and could be applied in healthy aging contexts. Eight KB elements were detected in the approaches though not all approaches incorporated all elements. The underlying conceptual guidance for KB approaches varied. Specific KB frameworks were referenced or developed for nine KB approaches while the remaining six cited more general KT frameworks (or multiple frameworks) as guidance.

**Conclusions:**

The KB approaches that we found varied greatly depending on the context and stakeholders involved. Three of the approaches were explicitly employed in the context of health aging. Common elements of KB approaches that could be conducted in healthy aging contexts focussed on acquiring, adapting, and disseminating knowledge and networking (linkage). The descriptions of the guiding conceptual frameworks (theories, models) focussed on linkage and exchange but varied across approaches. Future research should gather KB practitioner and stakeholder perspectives on effective practices to develop KB approaches for healthy aging.

**Electronic supplementary material:**

The online version of this article (doi:10.1186/s13012-016-0504-5) contains supplementary material, which is available to authorized users.

## Introduction

Developing a healthcare delivery system that is more responsive to the future challenges of an aging population is a priority in Canada [1–5]. The Canadian population aged 65 and over is expected to double over the next 25 years [6]. The rising number of people entering older age makes it likely that issues related to multi-morbidity, frailty, and chronic life-limiting illness will be a key challenge for healthcare systems in the next half-century. Healthcare providers and policy makers are expected to draw on research evidence in their provision of care or policy making [7–11]. Indeed, as acknowledged by the World Health Organization (WHO), understanding how to use research evidence (i.e., access, assess, and apply research knowledge to inform healthcare practices) is critical to maintaining healthy aging societies and addressing chronic diseases, housing, and community and social elements of aging [12].

The WHO recently released their World Report on Aging and Health, which defines healthy aging as “the process of developing and maintaining the functional ability that enables well-being in older age” [13]. The report provides a public health framework for healthy aging [13]. In addition, the report suggests key areas for action: (i) align health systems to the needs of older populations; (ii) develop long-term care (LTC) systems; and (iii) create age-friendly environments. To make progress in these areas of action, health system stakeholders (planners, providers, consumers) are encouraged to draw on research evidence to inform their decisions, be that the creation of new knowledge through the conduct of new research projects, or through the use of existing research evidence.

Increasing research use to improve practice is the purview of the field of Knowledge Translation (KT). KT refers to the practice and scientific inquiry that aims to ensure that stakeholders (i.e., care providers and recipients) are aware of and use research evidence to inform their health and healthcare decision-making [8], and its strategies are widely considered to help optimize the use of research evidence. One emerging approach to supporting the use of research is that of knowledge brokering (KB), a specific KT approach that includes making connections between researchers and decision-makers to facilitate the use of evidence in the promotion and provision of health and healthcare [14–17]. Knowledge brokers connect researchers and knowledge users to identify issues and problems for which solutions are required and facilitate the identification, access, assessment, interpretation, and/or translation of research evidence into local policy and practice [18]. We use the term approach because it is by definition “a way of dealing with a situation or a problem” (https://en.oxforddictionaries.com/definition/approach); approaches may entail multiple elements. Indeed, the roles of those involved described in KB approaches seem to vary greatly. Because KB is relatively new, many knowledge gaps continue to exist [16, 18], including understanding whether there are key KB elements that are consistent across various approaches. KB approaches as described in the literature [14, 15] appear to be adaptable to healthy aging contexts. Urquhart and colleagues [17] describe a specific KB approach that was applied in the area of healthy aging. In addition, many different KB models exist, including producer push models, linkage and exchange models, knowledge network models, and knowledge exchange team models [16, 17]. There have been increased calls for KT approaches, such as KB, to be guided by clearly stated theory or conceptual underpinning [19–23]. It is not clear whether there is a predominant conceptual framework or theory used to guide KB approaches. The terms conceptual frameworks, theories, and models are often used interchangeably, however conceptual frameworks tend to be broad and descriptive, while theories and models are more specific and better suited for testing and comparison [24]. Conceptual frameworks are used to guide practice and organize approaches. For simplicity, we have used the term “frameworks” to consistently refer to the conceptual underpinning from the KB approaches found in the literature.

We conducted a scoping review of the KB literature to better understand how to guide KB practice and organize approaches. The specific objective of the scoping review was to identify and describe KB approaches and the underlying conceptual frameworks (models, theories) used to guide the approaches that could be applied within the context of supporting healthy aging.

## Methods

Our scoping review approach was guided by that of Wilson and colleagues (2010). Our scoping review included the following steps: (1) identifying the research question; (2) searching for relevant studies; (3) selecting studies; (4) charting the data; (5) collating, summarizing, and reporting the results.

The following electronic databases were searched to identify studies for inclusion: PubMed, EMBASE, PsycINFO, EBM reviews (Cochrane Database of Systematic Reviews; ACP Journal Club; Database of Abstracts of Reviews of Effects; Cochrane Central Register of Controlled Trials; Cochrane Methodology Register; Health Technology Assessment; NHS Economic Evaluation Database), CINAHL, and Scopus. Searches in all databases covered the period from database inception until July 2014. With the support of a health sciences information specialist, a list of terms related to KB (knowledge brokerage, knowledge broker, knowledge brokering) was devised. The information specialist conducted a search using these terms (combined with the Boolean operator “OR”) in all fields of the databases listed above. Terms related to healthy aging were not used in the search as we wished to capture KB from a variety of contexts that could potentially be applied to health aging. No language or methodological limits were used. In addition, a search of the grey literature was conducted using Google and Google Scholar. The grey literature search used an expanded list of KB and KT terms (see Additional file 1 for the grey literature search strategy).

We considered inclusion for review in two stages:

1. First, the documents had to
  - be in English language
  - present an explicit and detailed description of a KB approach along with an explicit conceptual framework or model that was used to guide the KB approach
2. In documents that described a KB approach along with conceptual guidance, we included documents that:
  - described older adults as a recipient of care/services
    OR
  - described objectives to improve practice in health systems including long-term care (LTC) systems that could be adapted to meet the needs of older populations and/or to create age friendly environments.

We were inclusive and only excluded KB documents that were clearly not related to healthy aging contexts (as described by the WHO healthy aging report) [13] such as broad approaches related to the environment or those related to specific workplace organizational contexts.

Titles and abstracts were screened, according to the above inclusion criteria, independently by two reviewers. Reviewers met to discuss screening criteria after reviewing a number of documents to ensure we were consistent in interpreting the inclusion and exclusion criteria. When there were disagreements, reviewers met and came to consensus. When titles and abstracts were not excluded, the full article was obtained for further screening by two independent reviewers using the same inclusion/exclusion criteria. Disagreements were resolved by discussion. Documents that met the inclusion criteria were reviewed and details about the KB approach, target stakeholders, KB outcomes, and context were extracted. If more than one document described a single KB approach, we extracted data from all.

We were interested in how theory or conceptual frameworks/models were used to guide the KB approaches found in the literature. Therefore, we extracted information about underlying theories, models, and frameworks from the documents. The terms conceptual frameworks, theories, and models are often used interchangeably; however, conceptual frameworks tend to be broad and descriptive, while theories and models are more specific and better suited for testing and comparison [24]. Conceptual frameworks are used to guide practice and organize approaches. For simplicity, we have used the term “frameworks” to consistently refer to the conceptual underpinning from the KB approaches found in the literature.

Data extraction was carried out independently by two reviewers (DVE and KN) who then came to consensus about the data. A narrative synthesis of the KB approaches was done to explore and consider commonalities and underlying conceptual guidance. The descriptions of KB approaches were explored to identify common elements of KB, which we described according to the terminology from the CHSRF [14] and Ward [15] models.

## Results

**Fig. 1:**
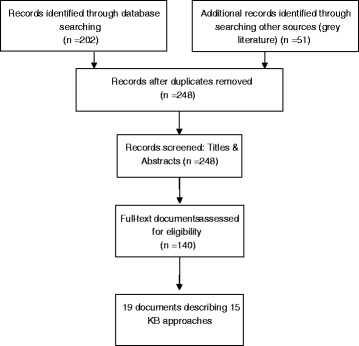
Inclusion of knowledge brokering (KB) approaches

The searches identified 248 unique references (see Fig. 1). Screening titles and abstracts resulted in the exclusion of 108 references. The remaining 140 full reports were screened to reveal 19 documents that described 15 distinct KB approaches (in two cases, multiple documents described the same KB approach) in sufficient detail. The documents were mostly from peer-reviewed journals (13 of 15) and were published/posted between 2003 and 2015, although 10 of 15 were produced since 2011. Two documents found in the grey literature searches were publically available reports issued by governmental agencies [14, 25].

Ten documents described seven distinct KB approaches that had been implemented in various healthcare or public health contexts [17, 18, 26–33]. The remaining nine documents described eight proposed KB approaches [14, 15, 22, 25, 34–38]. In some cases, the purpose of the documents was to develop and describe conceptual frameworks [14, 15, 22, 36, 38]. Table 1 provides a description of the 15 KB approaches in the final set of documents included in this scoping review.

**Table 1:** Knowledge broker approaches, target stakeholders, and outcomes described in relevant KB documents

Author, (year, jurisdiction)	KB approach implemented or proposed	Target Stakeholders	Outcomes evaluated or proposed?
Conklin (2013, Canada) [26, 27]	IMPLEMENTED: KB roles within a network (Seniors Health Research Transfer Network (SHRTN)) described: - coach/mentors to develop skills/capacity - knowledge translators (locate, appraise, create, package, disseminate knowledge) - developers of relationships/networks But note that the roles must be fluid and context dependent. Facilitating knowledge to action (KTA), communities of practice, and improved health services delivery for seniors.	• Caregivers • Policy makers • Researchers	PROPOSED: Proposed examining Knowledge to Action (KTA) processes and KB roles. No explicit KB outcomes evaluated
Dobbins (2009, Canada) [18, 28, 29]	IMPLEMENTED: 1. Access to a repository of high-quality research evidence (systematic reviews); 2. Tailored messages based on research evidence; 3. Individual knowledge brokers working one-on-one with decision-makers.	• Public health departments • Decision-makers • Researchers • Knowledge transfer practitioners	EVALUATED: The primary outcome assessed the extent to which research evidence was used in a recent program decision, and the secondary outcome measured the change in the sum of evidence-informed healthy body weight promotion policies or programs being delivered at health departments.
Gerrish (2011, UK) [30]	IMPLEMENTED: Description of advanced practice nurses disseminating information to clinical nurses through knowledge management (generating, accumulating, synthesizing, translating, disseminating research) and promoting the uptake of knowledge (capacity building, problem solving, facilitating change).	• Advanced practice nurses • Clinical nurses	EVALUATED: Promoting the uptake of knowledge: capacity building, problem solving, and facilitating change. (Evidence-based practice)
Goering (2003, Canada) [31]	IMPLEMENTED: Dissemination of information from two organizations in partnership. This was done through joint presentations by researchers and the ministry to the decision-making bodies in an interactive forum that allowed decision-makers to ask questions and seek clarification on information presented Using a tiered approach to linkage and exchange: - Inter-organizational relationship - Interactive research designs (working together) - Dissemination - Policy formation	• Policy makers; • Administrators/managers; • Frontline clinicians; • Decision-makers	EVALUATED: The researchers found that the forum format increased dissemination of the research product, provided clarification on language use with research and it provided insight into more targeted research based on experts in the field.
Henderson, (2011, Australia) [32]	IMPLEMENTED: Description of trained community-based health workers or "Community Navigators" helping disadvantaged community members navigate the health system and to promote positive health. Community navigators were based on a lay cultural health worker model and they described their knowledge broker roles including:. - Broad knowledge acquisition to provide information and knowledge not only about health issues, but also about the broader social determinants of health, such as housing, employment, and education. - operate in culturally appropriate ways within sub-groups of the community, including taking on interpreter roles for medical visits - Build capacity in the community: “working with the community to facilitate empowerment”	• Citizens/community groups • Administrators, managers	PROPOSED: Do community navigators make a difference to health equity in “culturally and linguistically diverse” communities with low access to health services? Focus on: awareness of health/healthy lifestyles, capacity for communities to effectively manage their own health (to seek medical assistance and to be able to communicate with doctors).
Urquhart (2011, Canada) [17]	IMPLEMENTED: Introduction of Knowledge Broker within a large research team to facilitate an integrated knowledge translation approach. Brokering tasks encompassed all activities relating to team interactions, communications, networking, stakeholder engagement/interaction, and research synthesis and dissemination.	• Cancer/health services researchers (large research teams)	EVALUATED: 1. Facilitation of an integrated KT approach to research conduct. 2. Development of collaboration between the research team and external stakeholders, including other researchers.
Wahabi (2011, Kingdom of Saudi Arabia) [33]	IMPLEMENTED: Family medicine physicians trained in evidence-based medicine to enhance their abilities as knowledge brokers through (1) Debates, where teams were scored on (i) comprehensiveness of their research, (ii) critical appraisal and grading of the evidence used during the debate, (iii) adaptation of evidence to participants’ local context, and (iv) the quality of the communication skills used to articulate the evidence to non-medical end users. (2) KT presentations using the KTA framework (3) EBM knowledge sessions where participant learned about EBM and how they may enhance their EBM skills.	• Frontline clinicians; (physicians)	NO: No KB outcomes described
Armstrong, (2013, Canada) [34]	PROPOSED: “KT4LG” A knowledge translation intervention for public health decision-making in local government. The intervention was designed to be implemented by a Program Coordinator who would also provide a point-of-contact and act as a KB. 1. Provision of evidence summaries and additional individualized support, such as tailored messages. 2. Training in accessing research, assessing trustworthiness, and applying research evidence to local context. 3. Developing and implementing strategies that assist in the development of an organizational culture that supports evidence-informed decision-making (EIDM) within local governments.	• Administrators/managers (decision-making staff at local governments) • Policy makers	PROPOSED: Intervention not yet implemented. Intention to measure individuals’ confidence, skills, and access to research evidence and to assess changes in organizational culture for EIDM.
CHSRF (2003, Canada) [14]	PROPOSED: Describes common core skills of KB in possible approaches: evidence gathering, critical appraisal, mediation, imagination/intuition, communication, and listening	• Healthcare stakeholders broadly	NO: Challenges of evaluation noted because will be context specific. No evaluation guidance provided.
Catello (2015, Canada) [35]	PROPOSED: Proposes key competencies for nurses to be knowledge brokers: evidence acquisition, critical appraisal, evidence-based decision-making experience, and networking.	• Nurses	NO: No evaluation conducted or proposed.
Hammami (2013, Canada) [36]	PROPOSED: A knowledge transfer process in which knowledge brokers are at the core, as well as a series of five activities to facilitate the link between researchers and users: - acquisition - integration - adaptation - dissemination - creation of links	• Knowledge brokers in the health service field	NO: Suggest organizational climate has a direct positive impact on brokers’ knowledge transfer activities through autonomy granted to brokers
Lemire (2013, Canada) [25]	PROPOSED: Exchange is central and knowledge translation is filtered through a multitude of intermediary actors, such as knowledge brokers: through a number of KT approaches: - as part of the social system in KT (i.e., an exchange network supporting the production and transfer of knowledge), - as a credible messenger, - facilitators (between two groups), - as a strategy to promote relationship development.	• Managers • Decision-makers • Stakeholders • Public policy makers • Professionals (liaison officers) • Knowledge translation officers • Researchers or knowledge brokers	NO: No evaluation was conducted.
MacDermid (2009, Canada) [37]	PROPOSED: KB noted as one of multiple methods of KT. KB role is to link decision-makers with researchers, facilitating their interaction to work collaboratively on using evidence for decision-making. Trust and development of common ground are essential. Suggest that KB should be used when: changes in policy are needed and where common goals/outcomes can be achieved among policy makers, end users, and knowledge developers.	• Healthcare providers (particularly hand surgeons rheumatologists, and therapists)	NO: No outcomes and no evaluation but based on improving evidence-based practice.
Ward (2009, England) [15]	PROPOSED: Multiple knowledge brokering approaches advocated, including (i) knowledge management through active dissemination and knowledge creation (ii) linkage and exchange though relationship building and facilitation and (iii) capacity building though education and training activities	• General healthcare policy and practice stakeholders (not necessarily policy makers)	NO: No specific KB outcomes described. Suggest a broader, more process oriented approach based on the underlying principles and processes of transferring knowledge into action.
Ward (2012, England) [22, 38]	PROPOSED: Interactive problem solving approach: The intervention involves helping participants identify, refine, and reframe their key issues, finding, synthesizing and feeding back research and other evidence, facilitating interactions between participants and relevant experts and transferring information searching skills to participants. Three KB approaches advocated: (i) information management (helping teams find, package, and disseminate information), (ii) linkage and exchange (facilitating discussions between the teams and relevant experts), and iii) capacity building (helping teams develop their capacity to exchange knowledge into the future).	• Mental health practitioners	NO: No specific KB outcomes described. Focus was on process and framework development.

There were eight elements of KB that emerged from the literature (Table 2). The KB elements that were described in three or more KB approaches include: creating knowledge (knowledge production, generation) [15], acquiring knowledge (gathering evidence, searching, accessing) [14], assessing knowledge (critical appraisal of evidence) [14, 15], adapting/translating knowledge (tailoring, preparing messages for stakeholders) [15], applying knowledge (implementing evidence in practice) [15], disseminating knowledge (transferring knowledge to target users/stakeholders) [14, 15], linking/networking (connecting with others, developing relationships) [14, 15], and enhancing capacity (developing skills in target users/stakeholders) [15].

**Table 2:** KB elements in approaches described in relevant KB documents

Author, (year, jurisdiction)	KB elements								
	Creating knowledge	Acquiring knowledge	Assessing knowledge	Adapting/translating knowledge	Synthesizing knowledge	Applying (implementing) knowledge	Disseminating knowledge	Linkage/networking	Enhancing capacity
Conklin, (2013, Canada) [26, 27]	+	+	+	+	–	–	+	+	+
Dobbins, (2009, Canada) [18, 28, 29]	–	+	–	+	+	–	+	–	–
Gerrish, (2011, England) [30]	+	+	–	+	+	+	+	–	+
Goering, (2003, Canada) [31].	–	–	–	–	–	+	+	+	–
Henderson, (2011, Australia) [32]	–	+	–	+	–	–	+	+	+
Urquhart, (2011, Canada) [17]	–	–	–	–	+	–	+	+	–
Wahabi, (2011, Kingdom of Saudi Arabia) [33]	–	+	+	+	–	–	+	–	–
Armstrong, (2013, Canada) [34]	–	+	+	+	–	+	+	+	+
CHSRF, (2003, Canada) [14]	–	+	+	–	–	–	+	+	–
Catello, (2015, Canada) [35]	–	+	+	–	–	–	–	+	+
Hammami, (2013, Canada) [36]	–	+	–	+	+	–	+	+	–
Lemire (2013, Canada) [25]	+	–	–	+	–	–	+	+	–
MacDermid, (2009, Canada) [37]	–	–	–	–	–	–	–	+	+
Ward, (2009, England)	+	v	–	+	–	+	+	+	+
Ward, (2012, England) [22, 38]	–	+	–	+	–	–	+	+	–

### KB approaches

The KB approaches implemented or proposed differed based on stakeholders and desired outcomes (Table 1). However, there were common elements that were described in a majority of approaches. Disseminating knowledge was an element mentioned in 13 of 15 KB approaches [14, 15, 17, 18, 22, 25–34, 36, 38]. Linking/networking was also mentioned in 13 of 15 KB approaches [14, 15, 17, 18, 22, 25–29, 31, 32, 34, 36, 38]. In addition, adapting/translating knowledge was noted in 10 of 15 approaches [15, 18, 22, 25–30, 32–34, 36, 38].

Acquiring knowledge was an element described in 10 of 15 KB approaches [14, 15, 18, 22, 25–30, 32–36, 38]. Enhancing capacity was mentioned in seven approaches [15, 26, 27, 30, 32, 34, 35, 37]. The remaining elements assessing knowledge, synthesizing knowledge, creating knowledge, and applying knowledge were mentioned less often (Table 2).

### Target stakeholders

Three of the approaches included in the review were used in the context of aging [17, 26, 27, 30] and considered clinicians, program administrators, and caregivers as target stakeholders. Otherwise, nine of the other 12 KB initiatives we reviewed considered specific healthcare settings and targeted decision-makers, practitioners, and patient stakeholders. Three other approaches focused on public health decision-makers. As such, the target stakeholders relevant to healthy aging addressed in these 15 KB approaches varied and included policy makers, nurses, researchers, community members, health services managers/administrators, and other knowledge brokers.

### KB outcomes

Outcomes specifically related to the KB approaches were reported or proposed in seven of the 15 approaches [17, 18, 22, 26, 27, 29–32, 34]. Outcomes reported were disparate and included measures of KB skills/role [26, 27, 34], research use/uptake/awareness [18, 22, 29, 30, 32], KT process [17, 26, 27], and practice change [18, 22, 29, 30, 32]. Other documents described an approach to KB but did not report on outcomes [14, 15, 22, 25, 33, 35–38].

### Conceptual underpinning for KB

Table 3 shows the conceptual frameworks or models that guided the KB approaches described. Most projects (i.e., 9 of 15) described specific KB frameworks [14, 15, 17, 22, 30, 31, 35–38], while the remaining six linked KB approaches to more general KT models [18, 22, 25–27, 29, 32–34].

**Table 3:** Conceptual frameworks (models, theories) noted in relevant KB documents

Author, (year), [jurisdiction]	KB conceptual framework, model, or theory (adopted, developed, or referenced)	Context
Conklin, (2013, Canada) [26, 27]	Adopted: the “linkage and exchange” model (CHSRF 2003) and the Promoting Action on Research Implementation in Health Services (PARIHS) framework (Kitson 1998; Kitson 2008). Also notes Ward (2009a) models as descriptive of KB roles	Health services delivery: communities of practice
Dobbins, (2009, Canada) [18, 28, 29]	Adopted: linkage and exchange model (CHSRF 2003; Lomas 2007) along with the framework for dissemination and utilization of research evidence for health care policy and practice (Dobbins 2002).	Public health
Gerrish (2011, England) [30]	References: linkage and exchange model (CHSRF 2003) and Ward (2009a) models	Healthcare
Goering (2003, Canada) [31]	Developed: A linkage and exchange framework that conceptualizes four tiers (inter-organizational relationship, interactive research projects, dissemination, and policy reform). Draws on Huberman 1994, Lomas 2000, Lavis 2002.	Public health: policy
Henderson (2011, Australia) [32]	Developed: the Community Navigators Model drawing on Lay cultural health worker model (Henderson et al. 2010), Community health worker approach (Lewin et al. 2007).	Healthcare, public health
Urquhart (2011, Canada) [17]	References and adopts: the CHSRF (2003) linkage and exchange model	Healthcare, health services
Wahabi (2011, Kingdom of Saudi Arabia) [33]	References: the Knowledge to Action framework (Graham 2007) as the basis for developing KB skills.	Healthcare
Armstrong (2013, Australia) [34]	Developed: KT logic model based on Bowen and Zwi (2005): Knowledge Translation for Local Government (KT4LG). A process model designed to guide a KT intervention with KB as coordinator.	Public health
CHSRF (2003, Canada) [14]	Developed: linkage and exchange model. The basis of the model is in knowledge management and the spreading of ideas leading to innovations. KB are intermediaries—linking and promoting exchange.	Healthcare
Catello (2015, Canada) [35]	References: linkage and exchange models (Lomas 2007, Ward 2009, Lavis 2013) as well as capacity development (Dobbins 2009, Robeson 2008, and Ward 2009).	Healthcare
Hammami (2013, Canada) [36]	Developed: a new exploratory framework based on KBs’ knowledge transfer activities: acquisition of new knowledge, integration of new knowledge, adaption of research results, dissemination of research, creating links between researchers and users. Multiple KT models were considered	Health services delivery
Lemire (2013, Canada) [25]	Developed: a process framework to guide dynamic KT approaches. KB role (called intermediaries) is considered central to the KT process. Conceptual basis for KB is not explicitly described.	Public health
MacDermid (2009, Canada) [37]	References: KB intervention description based on linkage and exchange (CHSRF 2003; Lomas 2007)	Healthcare
Ward (2009, England) [15]	References: three frameworks from Oldham and McLean (1997) to describe the functions of brokering: (1) The knowledge system framework (creation, diffusion, and use of knowledge); (2) transactional framework (interface between “creators” and “users” of knowledge); (3) social change framework (enhancing access to knowledge or capacity building)	Healthcare
Ward (2012, England) [22, 38]	Developed: based on sociological frameworks of diffusion and innovation (van de Ven 1999; Rogers 2003) a single conceptual framework of knowledge exchange (with KB as central component) with five loosely defined components: (i) problem identification and communication, (ii) analysis of context, (iii) knowledge development and selection, (iv) knowledge exchange activities/interventions, (v) knowledge use	Healthcare: mental health

The “linkage and exchange” model (created by the Canadian Health Services Research Foundation, now known as Canadian Foundation for Healthcare Improvement) (CHSRF 2003) was most often cited as the guiding framework for KB approaches [15, 17, 30, 31, 36, 37]. The CHSRF model was used either as the conceptual guidance for a KB approach or as the basis for developing new conceptual frameworks.

General KT models were noted as guiding three of the KB approaches [22, 26, 27, 33, 38]. Conklin et al. [26, 27] drew upon the PARiHS framework [39, 40] to guide the evaluation of the KB approach, while also using the CHSRF model [14]. Wahabi et al. [33] referred to the Knowledge to Action model [41] to guide a training approach for family physicians. In the development of a knowledge exchange model, Ward et al. [22, 38] and Armstrong [34] were influenced by diffusion of innovations [42, 43].

Nearly half of the KB approaches we examined were guided by multiple frameworks [15, 22, 26, 27, 31, 32, 34–36, 38]. Frameworks or models related to organizational learning [36], community navigators [32], and capacity development [15, 18, 35] were included along with the CHSRF and general KT models.

## Discussion

This scoping review was undertaken to find and describe existing knowledge on KB approaches along with their components and guiding conceptual frameworks. More specifically, we sought to identify KB approaches that could be applied in the context of healthy aging research as part of a larger research study. We found 15 distinct KB approaches providing details about broker activities, roles, skills, and competencies as well the underlying conceptual basis for the approaches. The goal of our review was not to evaluate the effectiveness of KB but rather to summarize the various KB approaches and conceptual guidance. We discuss the insights and implications of these scoping review findings now, first with respect to the elements and conceptual guidance of KB approaches, then in terms of supporting healthy aging.

### KB approaches and conceptual guidance

The KB approaches found in our review varied depending on the context and stakeholders involved. Not surprisingly, a majority of KB approaches described elements of disseminating knowledge and linking/networking, as well as acquiring and adapting knowledge. However, not one KB element was described in all of the approaches. The diversity of KB approaches was also described in recent literature reviews [16, 44]. Bornbaum and colleagues [16] reported that descriptions of KB roles consisted of multiple tasks that could be classified as knowledge management, linking agents, or capacity building. This is consistent with our findings of elements related to accessing, adapting, and disseminating knowledge as well as linking/networking. However, we did not find as much emphasis on capacity building in our review.

The goal of our review was not to evaluate the effectiveness of KB but rather to consider how KB is conducted. As such, our review was not limited to evaluation studies. In those studies that included evaluation, we noted a variety of study designs including a randomized controlled trial (18, 28, 29), multiple case study designs (26, 27, 30), and qualitative phenomenological analysis (32). We also explored the conceptual guidance of KB approaches. While there is no evidence that knowledge translation approaches guided by theory are more effective than those that are not [45, 46], there is support for using conceptual frameworks (models, theories) to direct approaches [19–23, 47].

We found that the “linkage and exchange” model from CHSRF [14] was the predominant guiding framework reported in the KB approaches we reviewed. General KT frameworks such as the Knowledge to Action model [41] or PARHIS [40] were also used to guide some KB approaches. As well, the approaches we found were often informed by multiple frameworks. Using multiple frameworks appeared to allow the KB approach to address specific contexts and stakeholders to achieve specific outcomes. More research is required to determine if KB approaches guided by theory will have greater impacts [20, 45]. However, as we consider KB approaches for healthy aging, we agree that conceptual guidance is important [19, 20, 41, 47, 48].

### Building a KB approach for healthy aging

Concurrent to our focus on describing KB approaches and conceptual guidance was our focus on aging. Our conception of healthy aging was guided by the WHO report on aging and health [13]. Specifically, we considered the WHO’s key areas of action: (i) align health systems to the needs of older populations; (ii) develop long-term care (LTC) systems; and (iii) create age-friendly environments to represent the contexts for KB approaches [13]. Three of the KB approaches examined in this scoping review were actually used in the context of health aging [17, 26, 27, 30]. The remaining KB approaches were deemed applicable to the field of healthy aging because they were described as adaptable and flexible to context and target stakeholders.

The three KB approaches from healthy aging contexts were guided by linkage and exchange frameworks [14, 15] and were applied in healthcare or health service contexts. However, the approaches did not describe the same KB elements. The only common KB element across these approaches was disseminating knowledge to a variety of stakeholders including clinicians, caregivers, policy makers, and researchers. Urquhart et al. [17] describe an emphasis on linkage and networking whereas Conklin [26, 27] and Gerrish [30] also include descriptions of acquiring and adapting knowledge. Similarly, the remaining 12 KB approaches were variable in the KB elements employed, suggesting that the approaches may emphasize different elements depending on context and objectives. However, taken together, we see that the KB approaches consistently consider acquiring, adapting, and disseminating knowledge as well as linkage/networking. One brief example from Gerrish [30] highlights a KB (eldercare nurse specialist) providing training to staff about elder abuse. The KB considered (acquired) information from guidelines (national and local) and her own experience, as well as from the media (TV, newspapers). Synthesizing the evidence and adapting it for the specific audience, the training (dissemination) was to impact on practice (application and enhancing capacity). The KB approach could be adapted for many contexts.

The findings from this scoping review can inform health system planners whose focus is on mobilizing knowledge to support healthy aging. The literature we reviewed presents a variety of approaches that can be adapted to the context of healthy aging, and within these approaches, we categorized eight KB elements that were described across the documents: creating knowledge, acquiring knowledge, assessing knowledge, adapting/translating knowledge, applying knowledge, disseminating knowledge, linking/networking, enhancing capacity. All of the elements were not applied in a single approach, which speaks to the flexibility and adaptability of KB approaches designed for specific contexts and outcomes. However, moving forward to design a KB approach for healthy aging, we suggest that all elements should be considered.

For instance, the evidence to support healthy aging practices will come from a broad range of research areas (e.g., gerontology, health services, geriatrics, primary care, public health, palliative care); therefore, tailoring the KB elements related to creating, acquiring, assessing, and synthesizing knowledge will be important and potentially challenging [14, 18, 22, 29, 30]. Given the aging population and increased global attention towards healthy aging [13, 49], we anticipate that the volume of relevant research will grow exponentially. Accordingly, close attention to target audiences’ needs and priorities is required in gathering and synthesizing high-quality research to support the practices and policies needed for healthy aging.

Beyond acquiring and synthesizing research evidence, and given the breadth of potential target stakeholders that will be involved in healthy aging practice and policy, the KB elements of adapting/translating and disseminating knowledge will also be essential [18, 22, 25–29, 32, 34, 38]. According to the WHO recommended actions, stakeholders from healthcare and LTC will be primary targets. Similar to other areas of health, the target stakeholders are wide ranging including administrators, healthcare providers, community providers, family members (caregivers), as well as policy makers at local and national levels. Moreover, the focus on creating age-friendly environments [13] suggests that in addition to the stakeholders noted above, there would be a need to connect with municipal, provincial/state, and a broader range of care providers. This is a broader audience than would be required for disease-specific KB and requires a level of connection (networking) across stakeholder groups from a range of geographical locations. KB approaches will have to consider the broad range of stakeholders and their diverse research and networking needs. Dissemination will need to be broad ranging and creative to effectively reach the stakeholders.

Perhaps the most important element of a KB approach, linkage, and network building will require much attention [14, 17, 25, 31]. The breadth of the potential stakeholders alone will be daunting to consider. Connecting, building, and maintaining relationships with multiple stakeholder groups will be time consuming and resource intensive. Linkage and networks are keys to the success of dissemination and essential to the element of capacity building [15, 26, 27, 30, 32]. Given the increases expected in the aging population, it will be vital that the KB approach build capacity to ensure that the effective practices and policies result in the changes desired. The “linkage and exchange” model [14] or many underlying aspects of the model (knowledge exchange and relationship elements) were a central part of all of the KB approaches in this review. The KB elements from the literature could fit within the concepts of linkage and exchange and therefore we propose that we use this as a guiding model for the KB approach for healthy aging.

### Strengths and limitations

A particular strength of this scoping review was that we sought and described KB approaches along with the theory/conceptual frameworks that guided them. Doing so allowed us to go beyond simply describing KB roles and activities and consider the broader approach and how it was guided. In addition, we described the KB approaches according to elements that were described in the literature. Considering KB elements can allow for the description of KB with less emphasis on the competencies and attributes of brokers. While broker talents are important to consider, we believe that KB approaches could be developed based on theory and the relevant elements based on the context of interest.

It is possible that we did not capture all KB approaches available in the literature, despite searching both peer-reviewed and grey literature sources. The literature on KT and KB are challenging to search with over 100 terms that can be used to describe similar KT practices, including KB [50]. We worked with an information specialist to develop parsimonious searches that were guided by the principles described by McKibbon et al. [50]. We did not limit our searches with terms related to aging because we wanted to capture a broad variety of KB approaches that could potentially be applied to healthy aging contexts. In addition, our inclusion criteria required a description of theory or conceptual guidance that resulted in a different set of documents from recent reviews of KB [16, 44, 48].

## Conclusions

The results from our scoping review reveal a number of documents that described KB approaches that can be considered relevant to healthy aging. Within these approaches, there was an emphasis on acquiring, adapting, and disseminating knowledge and networking (linkage). The elements of KB that most consistently characterized the studies in this scoping review suggest a good fit with the “linkage and exchange” conception of KB [14, 16, 17, 35]; and given the complex and multi-stakeholder nature of healthy aging contexts and practices, we suggest that linkage and exchange practices are likely to benefit KB efforts focused on supporting healthy aging.

The recent review by Bornbaum and colleagues [16] also reported on the variability of KB practice and did not find strong evidence of effectiveness. Therefore, future research should include gathering KB practitioner and stakeholder perspectives on effective practices and triangulate those data with the results of this scoping review to develop an evidence-informed KB approach for healthy aging. In addition, careful consideration should be given to appropriate conceptual guidance in developing KB approaches for healthy aging [20, 47, 48, 51–53].

## Additional file

Additional file 1: Grey literature search strategy. (DOCX 13 kb)

## Abbreviations

CHSRF: Canadian Health Services Research Foundation
KB: Knowledge broker
KT: Knowledge translation
LTC: Long-term care
PARiHS: Promoting Action on Research Implementation in Health Services
WHO: World Health Organization
