# Supplementary material for: Knowledge brokering for healthy aging: a scoping review of potential approaches
Source: Implement Sci. 2016 Oct 19;11:140. doi: 10.1186/s13012-016-0504-5 (PMC5070130; doi:10.1186/s13012-016-0504-5)
Supplement: Additional file 1: — Grey literature search strategy. (DOCX 13 kb) [file 13012_2016_504_MOESM1_ESM.docx]

**Knowledge brokering for healthy aging: A scoping review of potential approaches**

**Grey Literature Search Strategy**

**Search terms:**

Knowledge brokerage

Knowledge brokers
Knowledge brokering
Knowledge broker*
Knowledge translation
knowledge transfer
exchange of knowledge
knowledge synthesis
knowledge utilization
knowledge implementation
knowledge dissemination
knowledge diffusion
knowledge uptake
knowledge to action
"implementation of existing research knowledge"
knowledge-to-action
 knowledge to practice
knowledge mobilization
research linkage
research utilization
research dissemination
research into practice
research exchange
research uptake
applied health research
policy-relevant research
implementation science
coordinated implementation model
know-do-gap
"linkage and exchange"
"Diffusion of Innovation"
"bench to bedside"
 

For the grey literature search an information specialist (health sciences)was consulted who suggested using the google search function and google scholar. Each of the search terms listed above were entered into the search engine one by one.

The results obtained for each search term were reviewed to the point when no new relevant or useful information was emerging. Useful and/or relevant information was determined based on whether it presented models, frameworks, websites, policies, and/or articles specifically relating to the concept. Websites where definitions of concepts were provided were not included. Due to the similarity of the key terms, caution was applied to ensure no duplication of sources/documents occurred. Sources included contained website URLs to useful government and research organizations, research and information articles, frameworks/models, and limited policy related items.
